# Spermidine Revives Aged Sorghum Seed Germination by Boosting Antioxidant Defense

**DOI:** 10.3390/antiox14030349

**Published:** 2025-03-17

**Authors:** Yifan Xing, Huan Zhang, Chunjuan Liu, Chang Liu, Yufei Zhou

**Affiliations:** College of Agronomy, Shenyang Agricultural University, Shenyang 110866, China; yfxing1996@163.com (Y.X.); zh12082025@163.com (H.Z.); liuchunjuan@syau.edu.cn (C.L.); liuchang@syau.edu.cn (C.L.)

**Keywords:** *Sorghum bicolor* L., spermidine, aged seed, multi-omics, ROS, antioxidants

## Abstract

Seed aging has adverse effects on agricultural production, mainly because seed vigor is inhibited. Spermidine can improve seed vitality and germination ability to a certain extent and is essential for plant growth and plant response to stress. This study explored how spermidine counteracted aging effects on sorghum seed germination through antioxidant metabolism regulation. Aged seeds showed decreased vigor due to heightened reactive oxygen species (ROS) and diminished antioxidants. Applying spermidine notably enhanced aged seeds’ germination and vigor by boosting antioxidant enzyme activity and curbing ROS. Integrated transcriptomic, proteomic, and metabolomic analyses demonstrated that the majority of differentially expressed genes following exogenous spermidine treatment in aged sorghum seeds were significantly enriched in pathways related to glutathione metabolism, phenylpropanoid, and flavonoid biosynthesis, resulting in increased expression of genes encoding peroxidase, chalcone synthase, and glutathione s-transferase. Exogenous spermidine facilitated the synthesis of peroxidases and glutathione transferases. Analysis of flavonoid pathway intermediates showed a notable increase in antioxidant metabolites like isoquercitrin, underscoring their role in oxidative stress resistance. This multi-omics strategy underscores Spd’s role in boosting aged seeds’ antioxidants, highlighting the molecular basis of seed aging and Spd’s rejuvenating impact.

## 1. Introduction

Sorghum (*Sorghum bicolor* L.), a staple crop in arid and semi-arid regions, is renowned for its resilience and adaptability to harsh environmental conditions, making it a cornerstone of sustainable agriculture [1,2]. However, poor storage conditions and prolonged storage time often lead to seed aging, which reduces seed vigor, germination rates, and seedling performance [3]. This decline is closely associated with the accumulation of reactive oxygen species (ROS), which cause oxidative damage to cellular components, including lipids, proteins, and DNA [4,5]. For instance, Su et al. [6] have indicated that during the germination of aged maize seeds, reduced activities of superoxide dismutase (SOD), ascorbate peroxidase (APX) and catalase (CAT) lead to a significant increase in the levels of reactive oxygen species (O_2_^−^) and malondialdehyde (MDA) that is a primary product of lipid peroxidation and serves as a widely recognized biomarker for oxidative damage. Excessive ROS accumulation impairs mitochondria integrity in aged seeds [7,8], while proteomic analysis has revealed a significant enrichment of antioxidant-related proteins such as phenylalanine ammonia-lyase (PAL) in aged oat seeds [9]. Consequently, maintaining seed vigor requires a balance between ROS generation and the antioxidant system, although the physiological mechanisms and pathways remain poorly understood.

Recent studies have highlighted the role of polyamines, particularly spermidine (Spd), in modulating antioxidant responses and enhancing seed vigor under stress conditions [10,11,12]. Spd influences gene expression, protein synthesis, and metabolic pathways, thereby contributing to the overall antioxidant capacity of seeds [11,13,14]. For example, soaking sweet maize seeds in a 0.9 mM Spd solution for 12 h promotes seed germination, improves vigor, and preserves cell membrane integrity in the seedlings [15]. Similarly, Spd application enhances the germination rate of white clover (*Trifolium repens* cv. Haifa) seeds under cold stress and improves seed vigor and the germination index [16]. Under high-temperature stress increases antioxidant enzyme activity while reducing ROS accumulation [17]. These findings suggest that Spd modulates antioxidant defense mechanisms and improves germination in various crop species, including aging rice seeds [11].

Despite these advancements, the molecular mechanisms underlying its effects on antioxidant metabolism in aged seeds remain unclear. Secondary metabolism, particularly phenolic metabolites produced through flavonoid metabolism, is crucial in oxidative defense [18]. Phenylpropanoid metabolites, an upstream component of flavonoid metabolism, are a key pathway for antioxidant synthesis and seed storage tolerance [19,20]. For instance, in aged Chinese cabbage seeds, flavonoid biosynthesis is the most significantly affected pathway, with decreased metabolites such as chlorogenic acid and kaempferol [21]. Liu et al. [22] also identified phenylpropanoid biosynthesis as a key pathway for antioxidant synthesis and seed storage intolerance. Thus, exploring how Spd enhances seed aging resistances by regulating the biosynthesis of secondary metabolite biosynthesis is essential.

Seed aging is a complex process involving intricate biological regulation networks. Multi-omics approaches, including transcriptomics, proteomics, and metabolomics, can elucidate the regulatory mechanisms of germination in aged seeds. For example, the ability of soybean seeds to resist aging stress is primarily governed by the phenylpropanoid metabolism pathway [23]. Similarly, glutathione metabolism and phenylpropanoid biosynthesis improve wheat seed vigor [24]. However, little is known about the multi-omics information of Spd on aged seeds. Consequently, integrating these approaches can provide a comprehensive understanding of the cellular changes during seed aging and the potential of Spd to modulate these processes.

The objectives of this research were to (1) evaluate the impact of artificial aging on the antioxidant metabolism and germination performance of sorghum seeds; (2) determine the effects of exogenous Spd on the antioxidant capacity and germination of aged seeds; (3) identify the key genes and metabolites involved in the Spd-mediated antioxidant response using multi-omics analysis; and (4) elucidate the molecular mechanisms by which Spd enhances the germination potential of aged sorghum seeds.

## 2. Materials and Methods

### 2.1. Experimental Design

The study was conducted in the Sorghum Research Laboratory of the College of Agriculture, Shenyang Agricultural University. The sorghum variety ‘jiza123’ used in this experiment was an experimental variety, which was provided by the Crop Resources Research Institute, Jilin Academy of Agricultural Sciences. All sorghum seeds were stored in a refrigerator at 4 °C. To induce artificial aging in sorghum seeds, we employed the controlled deterioration treatment (ODT) method [25]. Specifically, sorghum seeds were completely immersed in distilled water at 58 °C for 45 min. Subsequently, the seeds were dried at 25 ± 1 °C until they reached their original moisture content (approximately 12%). Only full and uniform sorghum seeds were selected and disinfected with 5% sodium hypochlorite solution for 3–5 min and washed with distilled water. Previous experiments showed that the best germination was obtained with 0.05 μM of Spd solution. In our study, spermidine trihydrochloride (purity ≥ 98%, purchased from Sigma-Aldrich, St. Louis, MO, USA) was used as the source of Spd. The Spd was dissolved in sterile distilled water to prepare a 0.05 mM solution. To match the physiological conditions of the natural germination environment of the seeds, the pH of the solution was adjusted to 6.5. The three treatments were Control (raw sorghum seeds that had not been aged were directly subjected to germination experiment), ACK (seeds that had been aged by CDT were not subjected to exogenous Spd), and A-Spd (seeds soaked in 0.05 μM Spd solution after aging were subjected to germination experiment). Fifty sorghum seeds were placed into each Petri dish, and each treatment was repeated three times. Germination assays were performed at 25 °C in the dark, and the incubator humidity was set to 50%. The number of germinated seeds was counted at 0 h, 12 h, 24 h, 36 h, and 48 h of germination, and the germination rate was calculated.

### 2.2. Antioxidant Enzyme Activity and Malondialdehyde Content (MDA)

Sorghum seeds (1 g) were ground with 10% trichloroacetic acid and 0.5% thiobaric acid in the presence of fine quartz sand. The homogenate was transferred to a 10 mL centrifuge tube and centrifuged at 4000× *g* for 10 min using a refrigerated centrifuge (Eppendorf Centrifuge 5430, Hamburg, Germany). The supernatant was used for the determination of the MDA content according to the method of Xia et al. (2015) [26]. Antioxidant enzymes were extracted from sorghum seeds using the method of Gogorcena et al. (1995) [27]. SOD activity was measured using the method of Beyer and Fridovich (1987) [28]. POD activity was determined by the method of Fielding and Hall (1978) [29]. CAT activity was measured according to the method of Chen et al. (2022) [30]. APX activity was determined according to the method of Amako et al. (1994) [31]. The final OD value was determined by a spectrophotometer (Thermofisher, Shanghai, China).

### 2.3. ROS Content

The content of H_2_O_2_ was determined using a method [32]. Briefly, 0.5 g of the sample was weighed and ground in 11 mL acetone. The mixture was then centrifuged at 12,000× *g* at 4 °C for 20 min. 0.5 mL supernatant was taken and added with 100 μL 10% (*v*/*v*) titanium tetrachloride solution and 200 μL concentrated ammonia solution. After a reaction period of 5 min, the mixture was centrifuged again at 12,000× *g* and 4 °C for 15 min. The supernatant was removed, and 3 mL of 1 M sulfuric acid was added to the precipitate to dissolve. The absorbance of the resulting solution was measured at 412 nm. The O_2_^−^ content was determined using the method of Lin et al. [33].

### 2.4. RNA Extraction

Take a fresh sample (0.5 mg) and grind it rapidly in liquid nitrogen using TRIzol Universal reagent for 5–20 s. Incubate the homogenized sample at room temperature for 15 min to fully dissociate nucleic acid-protein complexes. Centrifuge the sample at 4 °C and 15,000× *g* for 10 min, then collect 0.6 mL of the supernatant. Add 0.2 mL of chloroform to the supernatant, vortex vigorously for 15 s, and incubate at room temperature for 15 min. Centrifuge the mixture again at 4 °C and 15,000× *g* for 15 min. RNA remains primarily in the aqueous phase. Transfer approximately 500 μL of the aqueous phase to a new centrifuge tube, add an equal volume of 99% isopropanol, mix thoroughly, and incubate at room temperature for 10 min. Centrifuge the mixture at 4 °C and 15,000× *g*) for 10 min, then discard the supernatant. Wash the pellet with 1 mL of 75% ethanol (prepared with RNase-free ddH_2_O). Centrifuge at 4 °C and 15,000× *g* for 5 min, carefully decant the liquid without disturbing the pellet. Briefly centrifuge the remaining liquid and remove residual traces using a pipette, ensuring the pellet remains intact. Air-dry the pellet at room temperature for 2–3 min (avoid over-drying, as completely dried RNA is difficult to dissolve). Finally, dissolve the RNA by adding 30–100 μL of RNase-free ddH_2_O and pipetting repeatedly to ensure complete resuspension.

The gene ids and primer information used for fluorescence quantitative analysis are shown in Table 1.

### 2.5. Transcriptome Analysis

Sorghum seeds germinated for 48 h were used for multi-omics analysis. The experimental process of transcriptome sequencing included RNA extraction (using agarose gel electrophoresis, Qubit 2.0 fluorometer (Shanghai, China), and Agilent 2100 bioanalyzer (Shanghai, China) to accurately detect RNA concentration and integrity), RNA detection, library construction, and computer sequencing on Illumina platform. After library inspection, different libraries were pooled according to the target amount of data for sequencing. Before conducting data analysis, the quality of the reads was assessed using fastp [34], ensuring that only high-quality reads were used. The reference genome used in this project is Sbicolor_454_v3.0.1.fa, available at the following download address: https://phytozome-next.jgi.doe.gov/info/Sbicolor_v3_1_1 (accessed on 22 July 2022). The number of reads of each gene was counted according to the comparison results and the location information of genes on the reference genome. FPKM (Fragments Per Kilobase of transcript per Million fragments mapped) was used as an indicator to measure the level of transcript or gene expression. DESeq was employed to analyze the expression of differential genes between sample groups. With the screening criteria set at |log_2_Fold Change| ≥ 1, and FDR < 0.05.

### 2.6. Metabolome Analysis

The experimental procedure for metabolomics sequencing included grinding sorghum seeds to powder into 30 Hz for 1.5 min using a grinder (MM 400, Retsch, Haan, Germany). The powder was dissolved in 1.2 mL of 70% methanol. The mixture was vortexed for 30 s every 30 min for a total of 6 times. After centrifugation at 13,400× *g* for 3 min, the supernatant was aspirated, and the samples were filtered through a 0.22 μm microporous filter membrane and stored in the injection vial for UPLC-MS/MS analysis [35]. The data acquisition instrument system mainly included Ultra Performance Liquid Chromatography (UPLC, Agilet SB-CIB 1.8 μm, 2.1 mm × 100 mm) (SHIMADZU Nexera ×2, https://www.shimadzu.com.cn/ (accessed on 22 July 2022)) and Tandem mass spectrometry (Tandem mass spectrometry, MS/MS, Applied Biosystems 4500 QTRAP, http://www.appliedbiosystems.com.cn/ (accessed on 22 July 2022)). The mass spectrometry conditions were as follows: electrospray ionization (ESI) source temperature: 550 °C; ion spray voltage (IS) set to 5500 V (positive ion mode) or −4500 V (negative ion mode); ion source gas I (GSI), gas II (GSII), and curtain gas (CUR) set to 50, 60, and 25 psi, respectively; collision-induced ionization parameter was set to high.

The metabolite content data were processed using UV (unit variance scaling), and the heat maps were generated using the R software (version 4.2.3) Complex Heatmap package. The accumulation patterns of metabolites in different samples were analyzed by Hierarchical Cluster Analysis (HCA). Pearson’s Correlation Coefficient (r) served as an evaluation index of biological repeat correlation.

### 2.7. Proteome Analysis

The proteomic sequencing protocol consisted of sample extraction followed by separation using NanoElute HPLC with the NanoElute system. The mixed samples were separated by chromatography, and mass spectrometry data were collected by ddaPASEF mode using the timsTOF Pro mass spectrometer (version tims TOF Pro 2). The step established a suitable acquisition window for the diaPASEF acquisition method. The mass spectrometry data were then analyzed using the Dia-nn (v1.8.1) with the library free method for DIA mass spectrum data analysis. The search parameters included the Uniprot Sorghum bicolor_2022.07.13. fasta database (comprising 41380 sequences in total), with deep learning parameters to predict a spectrum library. The MBR option was checked to generate a spectrum library from DIA data, which was subsequently used to reanalyze the DIA data for protein quantification [36].

### 2.8. Statistical Analyses

One-way analysis of variance (ANOVA) with SPSS18.0 (SPSS Inc., Chicago, IL, USA) and Duncan’s multi-range method was used to compare the difference levels among the treatments, and the significance level was set at *p* < 0.05. All figures were produced using GraphPad Prism 8 software (Graph Pad software Inc., San Diego, CA, USA).

## 3. Results

### 3.1. Effect of Exogenous Spd Treatment on Germination Ability of Aged Sorghum Seeds

**Figure 1:**
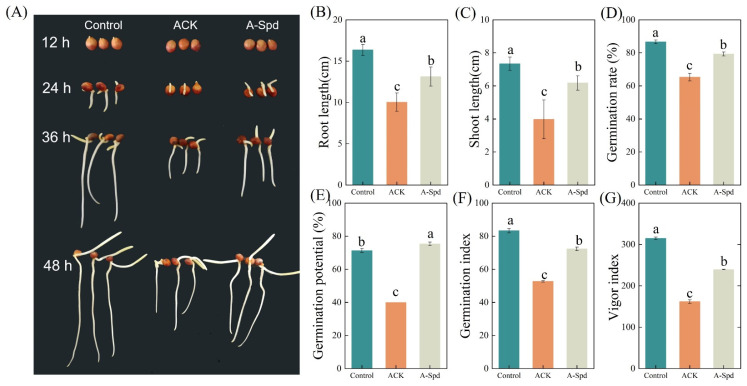
Effect of exogenous Spd treatment on germination of aged sorghum seeds. (**A**) Phenotypic. (**B**) Root length (cm). (**C**) Shoot length (cm). (**D**) Germination rate (%). (**E**) Germination potential (%). (**F**) Germination index. (**G**) Vigor index. Control, unaged sorghum seeds. ACK, aged sorghum seeds. A-Spd, Spd solution, and aged sorghum seeds. For each treatment, values were obtained from three biological repeats (*n* = 3). Different letters above the bars are statistically different by a least significant difference test (*p* < 0.05).

The germination performance of aged sorghum seeds was significantly reduced, but exogenous Spd treatment promoted germination (Figure 1A). Compared to the Control treatment, the germination rate of aged sorghum seeds significantly decreased by 24.3%. Conversely, Spd treatment increased the germination rate of aged seeds by 20.9%. Additionally, Spd significantly increased the germination potential, germination index, and vitality index of aged seeds. Compared to ACK treatment, these parameters of A-Spd treatment significantly increased by 88.2%, 37.4%, and 65.6%, respectively. Analysis of root and shoot length showed that seed growth of aged sorghum was significantly inhibited. However, compared to ACK, root and shoot length of A-Spd treatment significantly increased by 30.8% and 55.7%, respectively (Figure 1B).

### 3.2. Effect of Exogenous Spd Treatment on ROS Content and Enzyme Activities in Aged Sorghum Seeds

**Figure 2:**
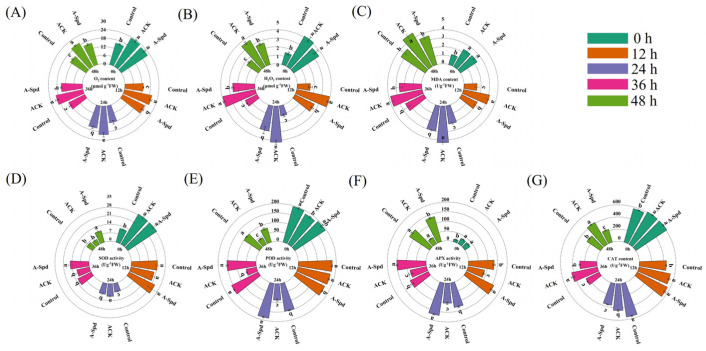
Effect of exogenous Spd treatment on O_2_^−^ and H_2_O_2_ contents of aged sorghum seeds during germination. (**A**) O_2_^−^ content (μmol g^−1^ FW). (**B**) H_2_O_2_ content (μmol g^−1^ FW). (**C**) MDA content (U g^−1^ FW). (**D**) SOD activity (U g^−1^ FW). (**E**) POD activity (U g^−1^ FW). (**F**) APX activity (U g^−1^ FW). (**G**) CAT activity (U g^−1^ FW). Control, unaged sorghum seeds. ACK, aged sorghum seeds. A-Spd, Spd solution, and aged sorghum seeds. For each treatment, values were obtained from three biological repeats (*n* = 3). Different letters above the bars are statistically different by a least significant difference test (*p* < 0.05).

Aging significantly increased the content of O_2_^−^ and H_2_O_2_ during sorghum seed germination. Compared to Control, the content of O_2_^−^ in ACK treatment increased by 47.8%, 51.7%, 65.2%, 53.3%, and 43.3% from 0 to 48 h, respectively (Figure 2A). The content of H_2_O_2_ increased by 180.4%, 155.7%, 249.1%, 151.3%, and 118.3% from 0 to 48 h, respectively. The A-Spd treatment significantly reduced the contents of O_2_^−^ and H_2_O_2_ in aged sorghum seeds from 12 h to 48 h. Compared to ACK treatment, the O_2_^−^ content of A-Spd treatment decreased by 12.0%, 20.9%, 35.8%, and 18.3%, respectively. The H_2_O_2_ content of A-Spd treatment decreased by 35.4%, 29.8%, 33.6%, and 26.0%, respectively (Figure 2B). Figure 2C showed that MDA content increased significantly after the germination of aged seeds and was significantly reduced by exogenous Spd treatment. Compared with the Control treatment, the MDA content of ACK treatment was significantly higher from 0 to 48 h. Compared with ACK treatment, MDA content in A-Spd treatment significantly decreased by 35.4%, 30.2%, 31.5%, and 28.5%, respectively, except for 0 h.

**Figure 3:**
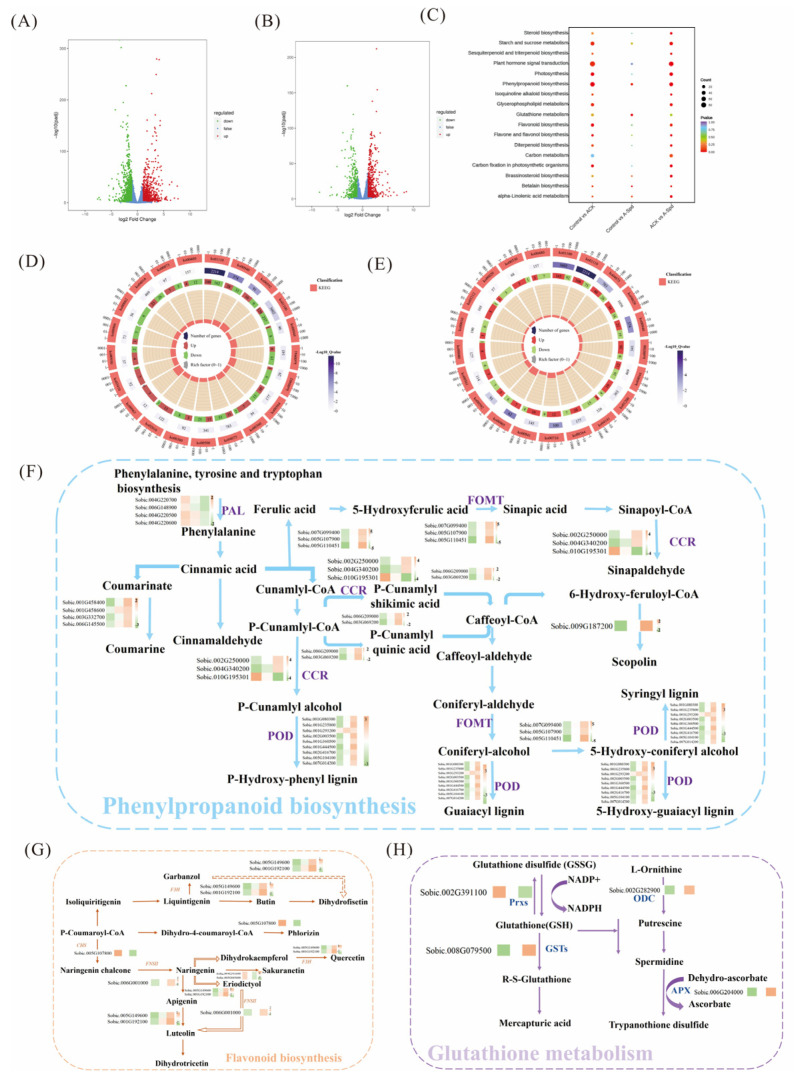
Transcriptome analysis of aged sorghum seeds in exogenous Spd. (**A**) is the volcano map of the Control vs. ACK comparison group. (**B**) is the volcanic map of the ACK vs. A-Spd comparison group. (**C**) Bubble maps of the three comparison groups. (**D**) is the enrichment map of KEGG pathway in the Control vs. ACK comparison group. (**E**) is the KEGG path in the ACK vs. A-Spd comparison group. (**F**) Pathway maps and heat maps of gene expression changes in phenylpropanoid biosynthesis. The sequence of differential gene heat maps is Control, ACK, and A-Spd. (**G**) Pathway maps and heat maps of flavonoid biosynthesis gene expression changes. (**H**) is the pathway map and heat map of glutathione metabolism gene expression change. Control, unaged sorghum seeds. ACK, aged sorghum seeds. A-Spd, Spd solution, and aged sorghum seeds. For each treatment, values were obtained from three biological repeats (*n* = 3).

Exogenous Spd treatment significantly increased SOD activity of aged seeds during germination, with increases of 45.2% and 113.0% at 36 h and 48 h, respectively, compared with ACK treatment (Figure 2D). Exogenous Spd treatment also significantly increased POD activity. Compared with the Control treatment, the POD activity of ACK treatment was lower from 12 to 48 h. In contrast, the POD activity of the A-Spd treatment was significantly higher than in the A-Spd treatment, except for 0 h (Figure 3E). Similarly, exogenous Spd treatment significantly increased APX activity, which was significantly decreased in ACK treatment compared with the Control treatment from 0 to 48 h. Compared with ACK treatment, the APX activity of A-Spd treatment was significantly higher except for 0 h (Figure 3F). Exogenous Spd treatment did not significantly increase CAT activity from 0–12 h. Compared with ACK treatment, CAT activity in A-Spd treatment was significantly decreased by 17.7%, 5.1%, and 34.7% from 24 to 48 h, respectively (Figure 3G).

### 3.3. Transcriptome Analysis of Aged Sorghum Seeds in Exogenous Spd

**Figure 4:**
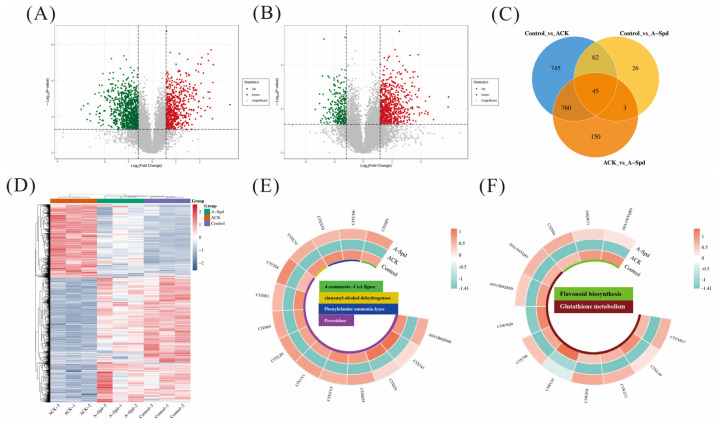
Proteome analysis of aged sorghum seeds treated with Spd. (**A**) The volcano map of the Control vs. ACK comparison group. (**B**) The volcanic map of the ACK vs. A-Spd comparison group. (**C**) Venn diagram of the three comparison groups (blue, Control vs. ACK; yellow, Control vs. A-Spd; orange, ACK vs. A-Spd). (**D**) The thermal map of proteins presenting the relative contents of proteins. (**E**) Thermogram of differential protein in phenylpropanoid biosynthesis pathway. (**F**) Thermogram of differential protein in flavonoid biosynthesis and glutathione metabolism. Control, unaged sorghum seeds. ACK, aged sorghum seeds. A-Spd, Spd solution, and aged sorghum seeds. For each treatment, values were obtained from three biological repeats (*n* = 3).

The differentially expressed genes (DEGs) of all comparison groups were combined for hierarchical clustering analysis. Compared with the Control group, the transcriptional level of ACK significantly decreased after germination, with the most pronounced reduction observed in genes related to antioxidant metabolism. In contrast, the transcriptional level of A-Spd showed a marked increase compared to the ACK, particularly in DEGs associated with antioxidant defense. The gene expression levels were similar to those in the Control treatment (Figure S1). The DEGs were analyzed across all comparison groups: Control vs. ACK, Control vs. A-Spd, and ACK vs. A-Spd, identifying a total of 2616 DEGs (Figure 3A,B). Among them, the numbers of up-regulated DEGs were 1176, 385, and 955, respectively, and the numbers of down-regulated DEGs were 1440, 122, and 633, respectively. In order to gain a deeper understanding of the functions of these DEGs, the metabolic pathways involved in the DEGs that responded to exogenous Spd after treatment were investigated through the kyoto encyclopedia of genes and genomes (KEGG) database annotations. The enriched metabolic pathways of Control vs. ACK and ACK vs. A-Spd comparison groups were mainly analyzed. In KEGG, the numbers of annotated DEGs in the Control vs. A-Spd and ACK vs. A-Spd comparison groups were 796 and 518, respectively. The number of DEGs enriched in each KEGG pathway was counted and sorted, with the top 21 pathways depicted in Figure 3D,E, and the top 17 pathways in Figure 3C. The present study found that 26 genes were significantly up-regulated in the control group of ACK vs. A-Spd in the phenylpropanoid biosynthesis pathway, which is one of the biological pathways for plant antioxidant production. The annotated *cinnamoyl-CoA reductase* (*CCRs*, *Sobic.004G340200.v3.2*), *PODs* (*Sobic.001G444500.v3.2*, *Sobic.001G235800.v3.2*, *Sobic.002G003500.v3.2*, *Sobic.007G014200.v3.2*, *Sobic.002G003500.v3.2*, *Sobic.001G293200.v3.2*, *Sobic.001G360500.v3.2*, *Sobic.005G104100.v3.2*) significantly up-regulated (Figure 4D). In the flavonoid biosynthesis pathway, six key genes were up-regulated, including *chalcone synthase* (*CHS*, *Sobic.005G107800.v3.2*), *flavonoid 3-hydroxylase* (*F3H*, *Sobic.005G149600.v3.2*), *flavone synthase II (FNSII*, *Sobic.006G001000.v3.2*), *CTs*(*Sobic.006G227000.v3.2*, *Sobic.006G227800.v3.2*) (Figure 4E). Additionally, four key genes in the glutathione metabolism pathway were up-regulated, including *glutathione S-transferase* (*GSTs*, *Sobic.008G079500.v3.2*), *Class III peroxidases* (*Prxs*, *Sobic. 002G391100.v3.2*), *ODC* (*Sobic.002G282900.v3.2*), and *APX* (*Sobic. 006G204000.v3.2*) (Figure 4F).

### 3.4. Proteome Analysis of Aged Sorghum Seeds Treated with Spd

The results of proteome analysis were similar to those of transcriptome. In the Control vs. ACK group, there were 1612 DEGs, with 1003 proteins down-regulated and 609 proteins up-regulated. In Control vs. A-Spd comparison group there were 958 DEGs comprising 270 proteins down-regulated and 699 proteins up-regulated. In the ACK vs. A-Spd comparison group, a total of 136 DEGs were identified, with 47 proteins down-regulated and 89 proteins up-regulated (Figure 4A,B). The proteomic cluster analysis showed that exogenous Spd had a significant effect on the aging of sorghum seeds. To further analyze the proteomic data, the protein response to exogenous Spd treatment versus aging treatment was visualized using the fold change (FC) and *p*-values of the volcano plot, which showed significant changes in aged seed proteins treated with Spd, mainly characterized by up-regulated expression (Figure 4D). The number of differential proteins contained in each KEGG pathway was counted, and the top nine pathways were plotted according to the number of DEGs enriched in the pathway (Figure 4E). To further elucidate the differential proteins related to antioxidant metabolism, three pathways were selected for detailed analysis consistent with the transcriptome findings. In the phenylpropanoid biosynthesis pathway, a total of 14 proteins were regulated (Figure 4F). These included 10 up-regulated peroxidases, two phenylalanine ammonolyses, one cinnamyl-alcohol dehydrogenase, and one 4-coumarate-CoA ligase. In the glutathione metabolism pathway, nine proteins were up-regulated, with C5YMG7, C5XU49, C5WZ12, C5WZ08, and C5WUI5 annotated as glutathione transferase. In the flavonoid biosynthesis pathway, three proteins were up-regulated: C5Z884, annotated as O-methyltransferase; A8QW51, annotated as O-methyltransferase 2(OMT2); and A0A194YQR9, annotated as flavonoid 3-hydroxylase (Figure 4F).

### 3.5. Metabolomic Response of Aged Sorghum Seeds with Spd

**Figure 5:**
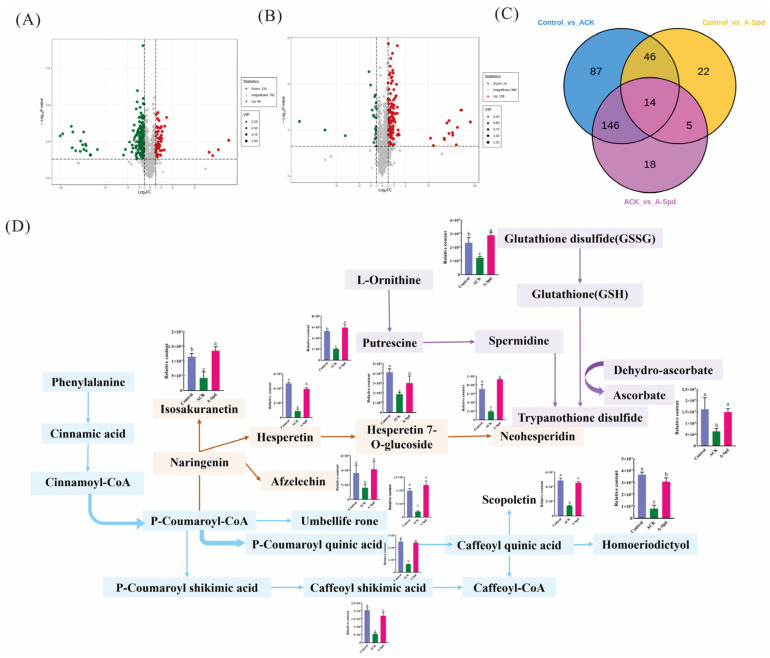
Metabolomic response of aged sorghum seeds with Spd. (**A**) The volcano map of the Control vs. ACK comparison group. (**B**) The volcanic map of the ACK vs. A-Spd comparison group. (**C**) Venn diagram of the three comparison groups. (blue, Control vs. ACK; yellow, Control vs. A-Spd; purple, ACK vs. A-Spd) (**D**) Changes in differential metabolite content in the KEGG pathway of phenylpropanoid biosynthesis (blue), flavonoid biosynthesis (yellow), and glutathione metabolism (purple). Control, unaged sorghum seeds. ACK, aged sorghum seeds. A-Spd, Spd solution, and aged sorghum seeds. For each treatment, values were obtained from three biological repeats (*n* = 3).

To evaluate how exogenous Spd treatment affected the expression of metabolites during germination of aged seeds, Figure 5A,B detected 293 significantly different metabolites in the Control vs. ACK comparison group by metabolome, of which 233 were down-regulated and 69 were up-regulated. A total of 183 significantly differential metabolites were detected in ACK vs. A-Spd, with 27 down-regulated and 156 up-regulated. Additionally, 87 significant differential metabolites were detected in the Control vs. A-Spd group, with 45 down-regulated and 42 up-regulated. The Venn diagram showed the overlap between the three different comparison groups, as well as the unique and shared differences in metabolites among the three different comparison groups, showing a total of 14 up-regulated metabolites (Figure 5C). The significantly enriched metabolic pathways in the Control vs. ACK and ACK vs. A-Spd groups were flavonoid biosynthesis (ko00941), phenylpropanoid biosynthesis (ko00940), and glutathione metabolism (ko00480). The flavonoid biosynthesis pathway is downstream of phenylpropanoid biosynthesis. Further analysis of the intermediates in the flavonoid biosynthesis pathway revealed significant enrichment of antioxidant secondary metabolites, such as isosakuranetin, chlorogenic acid, homoeriodictyol, afzelechin, 5-O-Coumaroylquinic acid, 5-O-Caffeoylshikimic acid, neohesperidin, and hesperetin, which are very important protective agents against oxidative stress. Figure 5D showed that the content of these antioxidant metabolites decreased significantly after seed aging relative to the control treatment. In contrast, the relative content of antioxidants was significantly up-regulated in A-Spd treatment compared to ACK treatment. Compared with ACK treatment, A-Spd treatment significantly increased oxidized glutathione (GSSG) and ascorbate in the glutathione metabolic pathway (Figure 5D).

### 3.6. Exogenous Spd Treatment Increased the Relative Expression of Antioxidant Genes in Aging Sorghum Seeds

**Figure 6:**
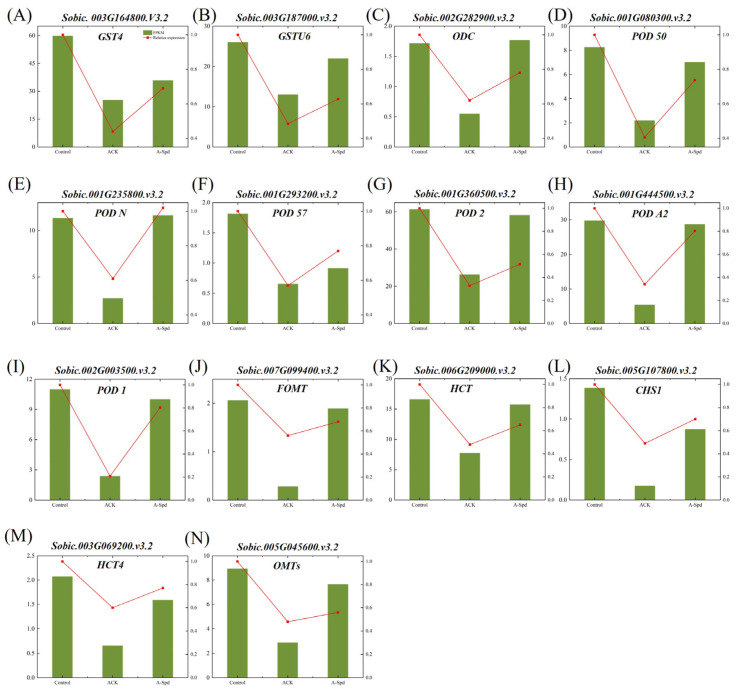
Effect of exogenous Spd treatment on relative expression of differentially genes in aged sorghum seeds. (**A**–**N**) transcript level of 14 selected DEGs analyzed by RT-qPCR (red line) and RNA-seq (bars). (**A**–**C**) Glutathione metabolism gene. (**D**–**J**) Phenylpropyl biosynthesis gene. (**K**–**N**) Flavonoid biosynthesis gene. Control, unaged sorghum seeds. ACK, aged sorghum seeds. A-Spd, Spd solution, and aged sorghum seeds. For each treatment, values were obtained from three biological repeats (*n* = 3).

Quantitative reverse transcription polymerase chain reaction (qRT-PCR) analysis was performed to confirm the expression patterns of differential genes, and the results showed that the expression of these genes assessed by qRT-PCR was basically consistent with the RNA-seq data. The expression of genes in glutathione metabolism, phenylpropanoid biosynthesis, and flavonoid biosynthesis pathways was significantly decreased in aged seeds. The relative expression levels of three key genes in the glutathione metabolism pathway (*Sobic.003G164800*, *Sobic.003G187000*, *Sobic.002G282900*) were significantly lower in ACK treatment than in Control treatment by 58%, 34%, and 61%, respectively (Figure 6A–C). Compared with ACK treatment, the expression of *Sobic.003G164800* and *Sobic.002G282900* genes were up-regulated by 64.3% and 74.2%, respectively. ACK treatment significantly reduced the relative expression of seven genes related to phenylpropanoid biosynthesis. In contrast, A-Spd treatment up-regulated the expression of five genes by 50.0%, 67.2%, 29.8%, 17.7%, and 25.0%, respectively, while the remaining two genes showed no significant difference (Figure 6D–J). The ACK treatment down-regulated the expression of four genes related to flavonoid biosynthesis, while the A-Spd treatment significantly increased their expression by 100%, 76.7%, 47.6%, and 16.7%, respectively (Figure 6K–N).

### 3.7. Multi-Omics Analysis of Antioxidant Metabolism in Aged Sorghum Seeds Treated with Exogenous Spd

**Figure 7:**
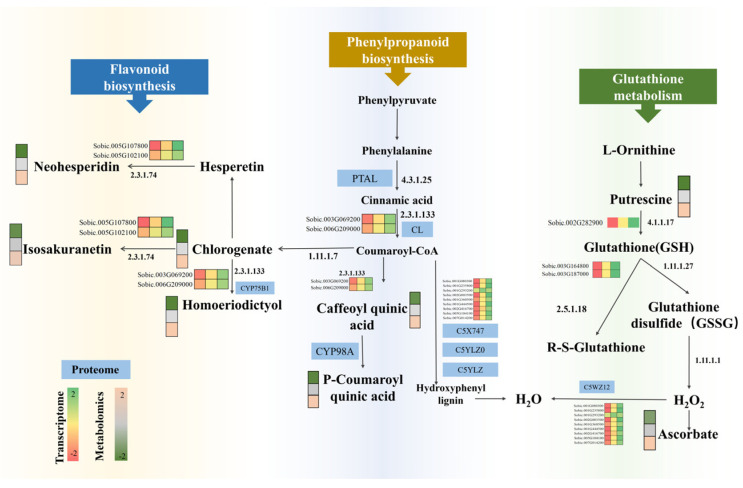
Multi-omics analysis of antioxidant metabolism in aged sorghum seeds treated with exogenous Spd. The sequence of differential gene heat maps is Control, ACK, and A-Spd.

Analysis of the pathways by which exogenous Spd treatment affected germination of aging sorghum seeds revealed that the combined analysis of differential genes, differential proteins, and differential metabolites formed a physiological regulatory network for phenylpropanoid biosynthesis, flavonoid biosynthesis, and glutathione metabolism (Figure 7). KEGG pathway enrichment analysis of differential metabolites and differential genes showed that phenylpropanoid biosynthesis, flavonoid biosynthesis, and amino acid synthesis pathways were significantly enriched. Specifically, four differential metabolites and 40 differential genes were involved in this metabolic pathway in the phenylpropanoid organism (ko00940), with relative expression levels of related metabolites significantly decreasing after aging but significantly increased after exogenous Spd treatment (Figure S2). Pathway enrichment analysis was performed on the associated proteome and transcriptome to determine the most important biochemical metabolic pathways in which differentially expressed genes and their corresponding differential proteins were involved. A total of 50 differential genes and their corresponding differential proteins showed the same expression trend after exogenous Spd treatment in seed aging (Figure S3), showing a highly significant correlation between transcriptome and proteome. According to the KEGG enrichment results, 34 proteins and 4 metabolites were enriched in the phenylpropanoid biosynthesis pathway in ACK vs. A-Spd. The pathway diagram of the phenylpropanoid biosynthesis network (Figure S4) showed that four metabolites (5-O-P-coumaroyl-quinic acid, chlorogenic acid (3-O-caffeoyl-quinic acid, 5-O-caffeoyl-shikimic acid, and umbellulone) were found to be in the central range, and the remaining proteins and metabolites were directly or indirectly related to them. In the flavonoid biosynthesis pathway (Figure S4), eight metabolites (hesperetin, isosakuranetin, homoeriodictyol, 5-O-coumaroylquinic acid, chlorogenic acid, hesperein-7-O-neohesperidin, 5-O-caffeyl shikimic acid, and afzelechin) were central.

## 4. Discussion

### 4.1. Aging-Induced Reduction in Sorghum Seed Vigor and Inhibition of Seed Germination

Seed germination traits are critical indicators of seed vigor and are affected by various environmental factors [37]. In this study, ACK exhibited notable declines in key phenotypic parameters, including germination rate, germination potential, germination index, and vigor index, compared with Control. This observation is consistent with previous studies across various species, such as cabbage [21], rice [22], oat [38], and maize [37]. During the aging process, seeds undergo a cascade of physiological and biochemical changes, including cell membrane damage, imbalances in the antioxidant system, and weakened metabolic activity [26]. At the cellular level, aging-induced cell membrane damage is characterized by increased membrane permeability and lipid peroxidation, both of which are key factors affecting seed vigor [39]. In addition, the decline in antioxidant enzyme activity in aged seeds leads to excessive accumulation of ROS [7], which further aggravates cellular oxidative damage and inhibits seed germination [21,40]. In this study, aging significantly increased MDA content and ROS levels during the germination of sorghum seeds (Figure 2). These findings highlight the close relationship between oxidative stress and reduced germination ability in aged seeds. At the molecular level, aging also affects gene expression patterns within seeds. The expression of genes involved in antioxidant defense, cell protection, and energy metabolism was down-regulated in aged seeds (Figure 3), which may be one of the molecular mechanisms leading to the reduction in seed vigor [22]. This downregulation may reduce the synthesis of antioxidant substances, thereby weakening the defense of seeds against oxidative stress.

### 4.2. Exogenous Spd-Mediated Enhancement of Seed Vigor and Antioxidant Defense in Aged Sorghum Seeds

Our study demonstrated that exogenous Spd significantly improved the germination performance of aged sorghum seeds. These observations suggest that Spd plays a crucial role in the seed germination process of plant seeds, especially under stress conditions, by improving plant tolerance to abiotic stress [41,42]. In this study, exogenous Spd treatment significantly increased the activity of antioxidant enzymes, including SOD, POD, and APX, which are essential for scavenging ROS and protecting cells from oxidative damage [43]. Furthermore, Spd treatment also reduced MDA content, indicating a mitigation of oxidative stress. Exogenous Spd treatment significantly up-regulated the expression of genes associated with antioxidant defense, such as those related to SOD, POD, and APX (Figure 2D–G). The upregulation of these genes helps to enhance the antioxidant defense system, thereby reducing oxidative damage and improving seed vigor under aging stress.

### 4.3. Spd-Induced Transcriptome Alterations and Its Role in Antioxidant Metabolic Pathways

Transcriptomic analysis revealed that exogenous Spd treatment significantly regulated the expression of genes involved in antioxidant metabolism, particularly in glutathione metabolism, phenylpropanoid biosynthesis, and flavonoid biosynthesis. Compared to the control group, an upregulation in the expression of key genes in these pathways was observed under Spd treatment, suggesting a pivotal role of Spd in gene expression regulation. Glutathione (GSH), the most predominant low-molecular-weight thiol compound within cells, plays a central role in the antioxidant defense system [44]. The knockout of genes encoding key enzymes in GSH biosynthesis, such as γ-glutamylcysteine synthase (GSH1) and glutathione synthase (GSH2), leads to embryonic lethality in Arabidopsis seeds [44]. The regulation of GSH synthesis and degradation occurs at transcriptional and post-translational levels, demonstrating the great flexibility of GSH metabolism in response to biotic and abiotic environmental challenges [45]. Glutathione S-transferases (GSTs) are a class of enzymes that catalyze reactions with GSH, facilitating the conjugation of GSH with various electrophilic endo- and xenobiotics, participating in detoxification and antioxidant defense, thereby helping plants cope with various biotic and abiotic stresses [46]. In addition to their role in the detoxification of xenobiotics, GSTs also function as isomerases, peroxidases, and transferases [47]. In this study, the expression of GST-related genes (*Sobic.001G318500*, *Sobic.001G249600*, *Sobic.001G318000*, *Sobic.001G319300*) was up-regulated, suggesting that exogenous Spd treatment-induced GSTs to enhance the ability of sorghum aged seeds to scavenge ROS through synergistic action with GSH.

Phenylpropanoid and flavonoid biosynthesis were also significantly enriched in Spd-treated seeds (Figure 3D,E). Phenylpropanoids are secondary metabolites widely involved in plant growth, development, and response to environmental stress [48]. It is not only involved in the metabolism of phenylalanine but also related to the synthesis of a variety of plant natural products, such as lignin and flavonoids [18]. For instance, Qiao et al. found that regulating the phenylpropanoid metabolic pathway could promote the synthesis of flavonoids and improve the antioxidant activity of soybeans [49]. In this study, antioxidant genes (*CCR* and *POD*) in phenylpropanoid biosynthesis were significantly up-regulated. Jia et al. [50] found that Spd may participate in the regulation of tomato’s adaptive response to salt stress by affecting the formation of fatty acid (FA) through PAL catalysis in the phenylpropanoid biosynthesis pathway, inhibiting the enzyme that produces free radicals, and having a good scavenging effect on H_2_O_2_. Thus, Spd plays an antioxidant regulatory role by inducing phenylpropanoid biosynthesis under stress conditions, thereby improving the tolerance to cope with seed aging. Flavonoids, downstream of phenylpropanoid biosynthesis, activate plant defense responses to biotic and abiotic stresses [51]. Similarly, exogenous Spd enhanced the accumulation of flavonoids compared with the control. CHS (chalcone synthase) is an enzyme involved in flavonoid biosynthesis and is the first rate-limiting enzyme in the flavonoid biosynthetic pathway [52]. In this study, exogenous Spd significantly up-regulated the expression of the CHS enzyme gene (*Sobic. 005G107800. v3.2*). QRT-PCR analysis showed the expression of CHS-related genes in flavonoid biosynthesis, such as *Sobic. 005G107800. v3.2*, was also significantly increased, indicating that exogenous Spd played a key role in regulating flavonoid biosynthesis, thereby enhancing the antioxidant defense ability of aging sorghum seeds.

### 4.4. Contribution of Proteomic Changes Induced by Spd to Enhanced Antioxidative Metabolism

The comparative proteomic analysis between ACK and Spd-treated aged seeds identified a substantial number of differentially expressed proteins, with a notable upregulation in response to Spd treatment. This upregulation was particularly evident in proteins associated with antioxidant metabolism, such as those involved in phenylpropanoid biosynthesis, flavonoid biosynthesis, and glutathione metabolism. The upregulation of these proteins suggests that Spd plays a crucial role in enhancing the antioxidant capacity of aged seeds, which is essential for their germination and vigor (Figure 4). A significant finding in this study was that Spd treatment markedly up-regulated the expression of proteins associated with antioxidant defense, such as glutathione S-transferases (GSTs) and peroxidases (PODs), which play a central role in alleviating oxidative stress [53]. The up-regulated expression of GSTs and PODs likely enhanced the ability of aged seeds to scavenge ROS, thereby protecting seeds from oxidative damage. This finding is consistent with the upregulation of corresponding gene expressions in the transcriptome data. Furthermore, the upregulation of these antioxidant proteins may be related to Spd-induced signal transduction pathways, such as the mitogen-activated protein kinase (MAPK) cascade, which plays a central role in plant responses to stress [54]. Meanwhile, the regulation of metabolic pathways by Spd treatment was also closely related to protein synthesis and metabolism. For example, the changes in protein expression were observed in phenylpropanoid biosynthesis and flavonoid biosynthesis pathways, with the key enzymes in these pathways, such as chalcones synthase (CHS) and flavonoid 3-hydroxylase (F3H) being up-regulated, potentially affecting the synthesis of metabolites 5-O-p-coumaryl quinic acid and chlorogenic acid [27,55]. The accumulation of these metabolites not only enhances the antioxidant capacity of seeds but may also act as signaling molecules involved in regulating gene expression and protein synthesis during seed germination [56,57].

The regulatory effects of Spd on protein synthesis and metabolism may be achieved by affecting post-translational modifications and protein stability [58]. For example, exogenous Spd may affect the stability and function of proteins by participating in the modulation of enzymes associated with protein phosphorylation [59]. This regulation may act in concert with the gene expression changes observed in the transcriptome data, collectively affecting protein synthesis and metabolism.

### 4.5. Metabolomic Adjustments Induced by Spd Contribute to Enhanced Antioxidative Metabolism

The metabolomic analysis in this study revealed significant effects of exogenous Spd on the metabolome of aged sorghum seeds, elucidating how Spd improved germination ability and stress resistance. By comparing the metabolic differences between aged seeds (ACK) and those treated with exogenous Spd (A-Spd), Spd markedly modulated metabolites related to antioxidant metabolism, particularly those in phenylpropanoid biosynthesis and flavonoid biosynthesis pathways. These metabolites include isosorbide, chlorogenic acid, homoerysipelol, afatechin, 5-O-p-coumaroyl-quinic acid, 5-O-caffeoyl-shikimic acid, neohesperidin, and hesperetin, all of which play key roles in antioxidative stress [60,61,62]. Compared to ACK treatment, A-Spd treatment significantly up-regulated the content of these antioxidant metabolites, indicating that exogenous Spd enhanced the antioxidant capacity of aged sorghum seeds by enhancing the biosynthesis of antioxidant metabolites, thereby promoting seed germination.

Metabolomic data also revealed significant effects of Spd treatment on flavonoid biosynthesis pathways in sorghum seeds. Flavonoids are not only potent antioxidants that activate plant defense responses to both biotic and abiotic stresses [63]. Our results are consistent with previous studies showing that increased levels of flavonoids can improve plant tolerance to salt stress. In the present study, the content of flavonoid metabolites in aged seeds significantly increased after exogenous Spd treatment, which may be related to elevated antioxidant activity during seed germination. In addition, Spd treatment affected metabolites related to energy metabolism and amino acid synthesis, which could improve the energy status and synthesis capacity of aged seeds, thus increasing their germination rate [64,65]. These findings highlight the potential role of Spd in regulating the metabolome of aged sorghum seeds in response to oxidative stress, providing a metabolomic rationale for Spd as a potential seed treatment agent.

### 4.6. Integrating Multiple Omics to Elucidate the Role of Spd in Improving Antioxidative Metabolism of Aged Sorghum Seeds

The integration of transcriptomic, proteomic, and metabolomic approaches has emerged as a powerful tool for dissecting the complex regulatory networks involved in biological processes, particularly under adverse conditions [66,67]. In this study, we employed a multi-omics strategy to elucidate the mechanisms by which exogenous Spd enhances the germination potential of aged sorghum seeds. Our results provide a comprehensive view of the transcriptional and metabolic changes that occur during seed aging and reveal the potential for Spd to modulate these processes, offering insights into the molecular mechanisms underlying the revitalizing effect of Spd on aged seeds.

Our transcriptomic analysis indicated that exogenous Spd treatment significantly modulated the expression of genes involved in antioxidant metabolism, particularly in the phenylpropanoid biosynthesis, flavonoid biosynthesis, and glutathione metabolism pathways [68]. The upregulation of genes in these pathways suggests that Spd may enhance the antioxidant capacity of aged seeds by promoting the synthesis of antioxidant compounds, which in turn could protect seeds from oxidative damage and improve germination.

Proteomic analysis further supported the transcriptomic findings, showing an upregulation of proteins associated with antioxidant metabolism in response to Spd treatment. This upregulation was particularly evident in proteins involved in phenylpropanoid biosynthesis, flavonoid biosynthesis, and glutathione metabolism. The increase in antioxidant protein content suggests that Spd enhances the seeds’ ability to detoxify ROS, thereby reducing oxidative stress and improving seed vigor. This aligns with the idea that polyamines can influence protein synthesis and stability, contributing to the overall antioxidant capacity of seeds [57].

Metabolomic adjustments induced by Spd were also significant, particularly in the phenylpropanoid and flavonoid biosynthesis pathways. Our data showed that Spd treatment led to an accumulation of antioxidant metabolites, such as isoquercitrin, chlorogenic acid, and kaempferol, which are crucial for managing oxidative stress [69,70]. These findings are supported by previous research highlighting the importance of phenylpropanoid metabolism in plant development and responses to environmental interactions [20]. The accumulation of these metabolites in response to Spd treatment indicated that Spd may enhance the seeds’ antioxidant defenses, thereby improving their ability to withstand aging-related oxidative stress and promoting germination.

## 5. Conclusions

**Figure 8:**
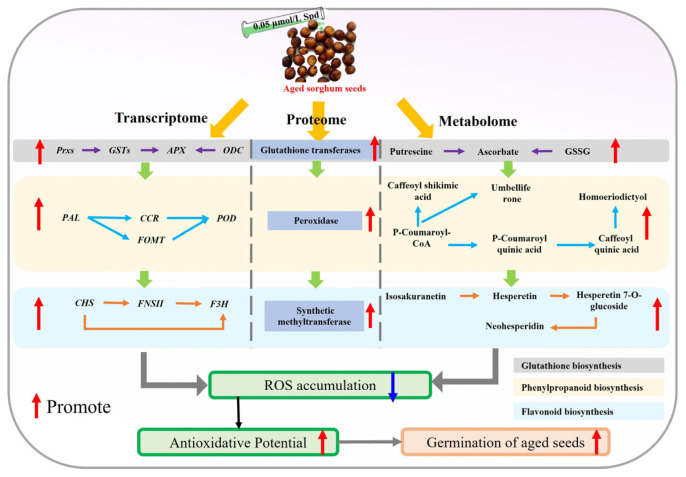
A flowchart of exogenous spermidine promoting germination of aged sorghum seeds.

This study demonstrated that exogenous spermidine (Spd) significantly enhanced the germination potential and antioxidant capacity of aged sorghum seeds through the modulation of key metabolic pathways (Figure 8). Our multi-omics approach revealed that Spd treatment up-regulated genes and proteins involved in phenylpropanoid biosynthesis, flavonoid biosynthesis, and glutathione metabolism, leading to increased levels of antioxidant metabolites such as chlorogenic acid. These changes collectively mitigated oxidative stress and improved seed vigor by enhancing antioxidant enzyme activities and scavenging ROS. The findings highlight the potential of Spd as a seed treatment agent to counteract the negative effects of aging, enhance germination, and improve stress tolerance in sorghum seeds, offering valuable insights for sustainable agricultural practices and germplasm conservation.

## Figures and Tables

**Table 1:** Primer used for qRT-PCR analysis.

Gene Name/Gene ID	Forward Primer	Reverse Primer	bp
Actin	CATTCACGAGACTACCTAC	GACGATGTTGCCATATAGA	180
*GST4/Sobic.003G164800*	CTGCCACTTCGGTTTCA	CGGCCATCACCTTCTGC	128
*GSTU6/Sobic.003G187000*	CGGCATGATGGTGAAGGC	GTCCAGGTACCCGGGCTC	157
*ODC/Sobic.002G282900*	GCCTCTACGGCTCGCTC	GTCGCCCACGCTCATCT	176
*POD50/Sobic.001G080300*	AGCTGCTGGCGGTCTTCA	ACTGCGCGTACTTGGGGT	168
*PODN/Sobic.001G235800*	TCGACAACAACTACTACAAGAAC	GGACCAGACGAAGTCACAGA	158
*POD57/Sobic.001G293200*	GGTCGCCTACTACGCCA	TCGGACACACCCACTTCT	194
*POD2/Sobic.001G360500*	CCGTTTTAGCCGCACTT	CAGCCTGGGGACACTTG	105
*PODA2/Sobic.001G444500*	ACGCTGGACAGGGGGTA	TCGACGTTGGTGTAGTAGTTG	128
*POD1/Sobic.002G003500*	TGGGCGCGTACAACAAGA	AGCAGTCCACGGAGAAGA	123
*FOMT/Sobic.007G099400*	TCAACTCAACATCCCCACT	AATACCTGATACAACAAGAAACC	139
*HCT/Sobic.006G209000*	GTGCCCAAGAAGAAAGCC	CAAGCGACGAGGAACATA	131
*CHS1/Sobic.004G107800*	ACGTCTTCCGTGGTCTGA	TGGCGATAACCTGTGGG	126
*HCT4/Sobic.003G069200*	TTGCCACAAACCGACCT	CTCCGCCAGCGACATCA	99
*OMTs/Sobic.005G045600*	AGAGAGATGAGCAAGAATGGA	CGAACACCTAAAACTGGTATGA	82

Note: bp, base pair. Tm value was 60 °C. GST4, glutathione S-transferase 4; GSTU6, glutathione S-transferase; ODC, ornithine decarboxylase; POD50, peroxidase 50; PODN, peroxidase N; POD57, peroxidase 57; POD2, peroxidase 2; PODA2, peroxidase N; POD1, peroxidase 1; FOMT, flavonoid O-methyltransferase; HCT, hydroxycinnamoyl transferase; CHS1, chalcone synthase 1; HCT4, Hydroxycinnamoyl transferase 4; OMTs, O-methyltransferase.

## Data Availability

Data are contained within the article.

## Supplementary Materials

The following supporting information can be downloaded at: https://www.mdpi.com/article/10.3390/antiox14030349/s1. Figure S1: cluster heatmap of all differentially expressed genes; Figure S2: combined analysis of metabolome and transcriptome; Figure S3: combined analysis of transcriptome and proteome; Figure S4: combined analysis of proteome and metabolome; Table S1: statistical table of sample sequencing data evaluation.
