# Supplementary material for: Spermidine Revives Aged Sorghum Seed Germination by Boosting Antioxidant Defense
Source: Antioxidants (Basel). 2025 Mar 17;14(3):349. doi: 10.3390/antiox14030349 (PMC11939325; doi:10.3390/antiox14030349)
Supplement: Supplementary file 1 [file antioxidants-14-00349-s001.zip › antioxidants-3487247-supplementary.pdf]

Supplemental Information

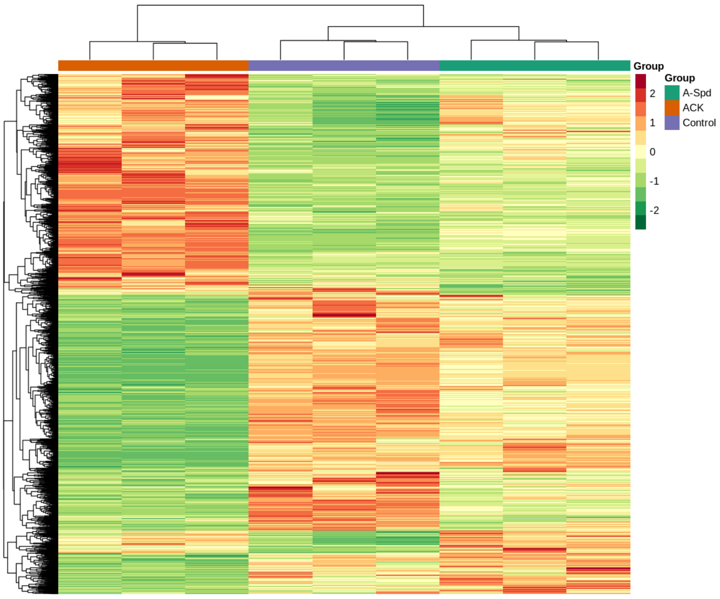

Figure S1. Cluster heatmap of all differentially expressed genes. Control, unaged sorghum seeds; ACK, aged sorghum seeds; A-Spd, Spd solution and aged sorghum seeds.

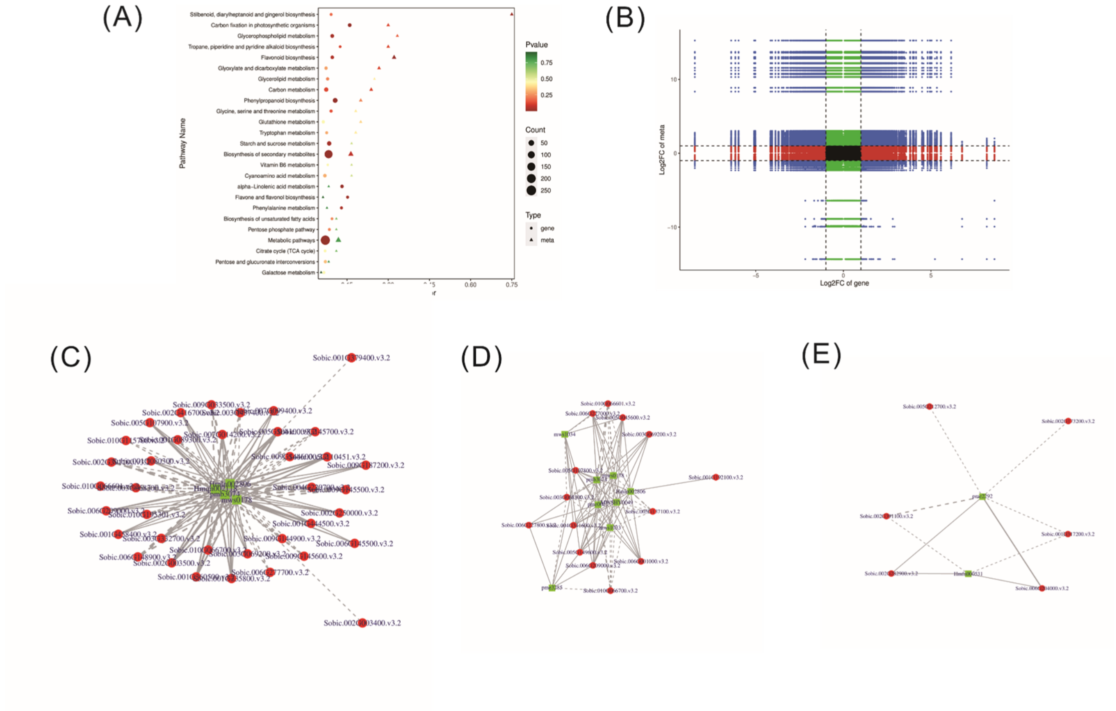

Figure S2. Combined analysis of metabolome and transcriptome. (A) KEGG enrichment analysis bubble map; (B) nine-quadrant diagram of correlation analysis; (C) ko00940 correlation network diagram; (D) ko00941 correlation network diagram; (E) ko00480 correlation network diagram. Control, unaged sorghum seeds; ACK, aged sorghum seeds; A-Spd, Spd solution and aged sorghum seeds.

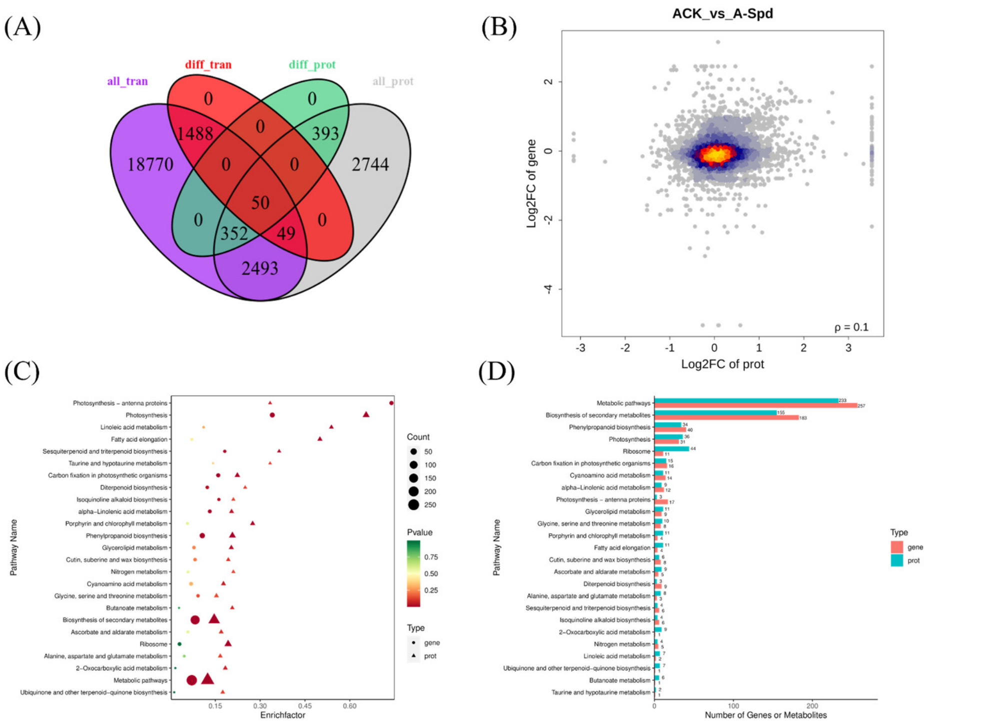

Figure S3. Combined analysis of transcriptome and proteome. (A) Venn diagram of differentially expressed proteins and genes (B) Scatter heat map of fold change of proteins and genes (C) KEGG enrichment analysis bubble plot (D) KEGG enrichment analysis bar plot. Control, unaged sorghum seeds; ACK, aged sorghum seeds; A-Spd, Spd solution and aged sorghum seeds.

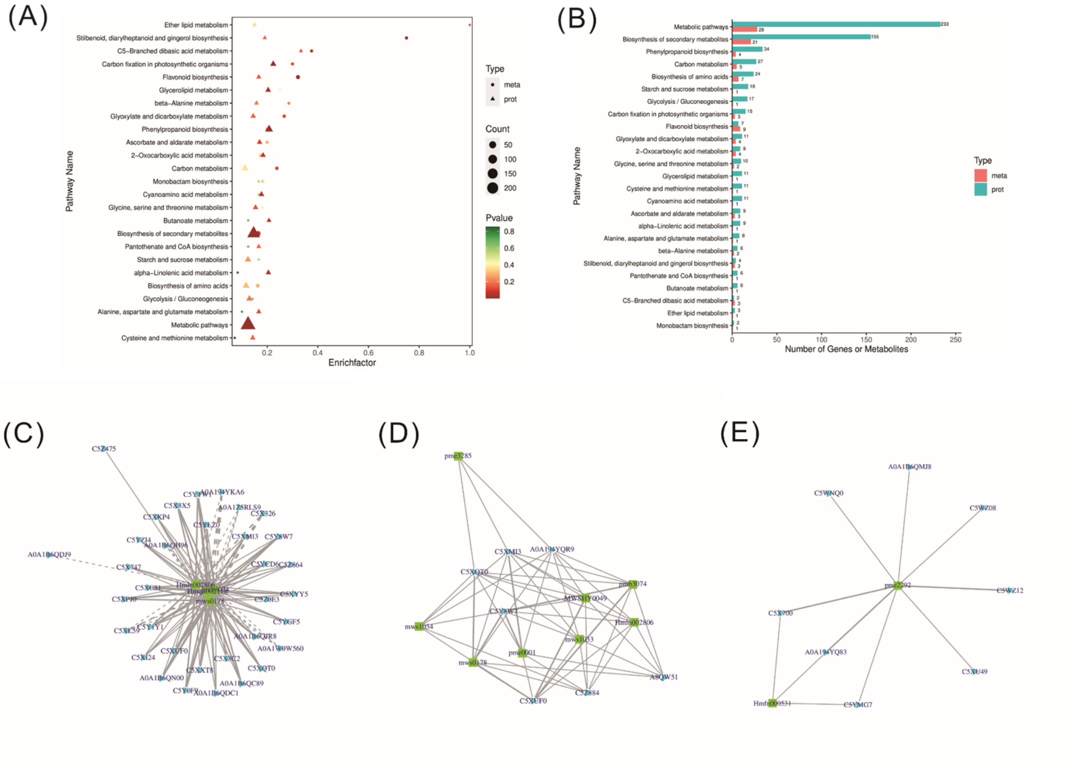

Figure S4. Combined analysis of proteome and metabolome. (A) KEGG enrichment

analysis bubble diagram (B) KEGG enrichment analysis bar diagram (C) ko00940 correlation network diagram; (D) ko00941 correlation network diagram; (E) ko00480 correlation network diagram. Control, unaged sorghum seeds; ACK, aged sorghum seeds; A-Spd, Spd solution and aged sorghum seeds.

Table S1. Statistical table of sample sequencing data evaluation

Sample	Raw Reads	Clean Reads	Clean Base (G)	Error Rate (%)	Q20 (%)	Q30 (%)	GC Content (%)
Control-1	50045328	49548494	7.43	0.03	98.00	94.20	58.26
Control-2	47544866	46996726	7.05	0.03	98.01	94.24	57.98
Control-3	53641410	53095648	7.96	0.03	97.98	94.15	58.59
ACK-1	49689832	49194768	7.38	0.02	98.11	94.44	57.15
ACK-2	47449972	46973514	7.05	0.03	97.98	94.09	56.64
ACK-3	50214170	49654198	7.45	0.03	97.86	93.85	57.14
A-Spd+1	62818810	61887534	9.28	0.03	97.79	93.96	58.04
A-Spd+2	45837696	45366526	6.80	0.02	98.10	94.41	57.40
A-Spd+3	130355126	128675330	19.30	0.03	97.61	93.51	58.16

Note: Control, unaged sorghum seeds; ACK, aged sorghum seeds; A-Spd, Spd solution and aged sorghum seeds.

Before data analysis, it is first necessary to ensure that these Reads are of high enough quality to ensure the accuracy of subsequent analysis. In this experiment, fastp was used for strict quality control of data, and clean reads were generated by removing paired reads with adapters, N content exceeding 10% of the base number of the read, ambiguous nucleotides and low-quality sequences. In this study, the percentage of Q30 base was higher than 93.51%, and the error rate of sequencing results was lower than 0.03%. Compared with the reference sorghum genome, the ratio of sequencing data ranged from 94.81% to 95.92%. The quality of the sequencing results was good enough for further analysis (Table S1).
